# Effects of Newer Antidiabetic Drugs on Endothelial Function and Arterial Stiffness: A Systematic Review and Meta-Analysis

**DOI:** 10.1155/2018/1232583

**Published:** 2018-12-04

**Authors:** Konstantinos Batzias, Alexios S. Antonopoulos, Evangelos Oikonomou, Gerasimos Siasos, Evanthia Bletsa, Panagiota K. Stampouloglou, Chara-Vasiliki Mistakidi, Marina Noutsou, Niki Katsiki, Periklis Karopoulos, Georgios Charalambous, Anastasia Thanopoulou, Nicholas Tentolouris, Dimitris Tousoulis

**Affiliations:** ^1^1st Department of Cardiology, Hippokration Hospital, National and Kapodistrian University of Athens Medical School, Athens, Greece; ^2^Diabetes Center, 2nd Department of Internal Medicine, Medical School, National and Kapodistrian University of Athens, Hippokration General Hospital of Athens, Athens, Greece; ^3^Second Department of Internal Medicine, Hippokration University Hospital, Aristotle University of Thessaloniki, Thessaloniki, Greece; ^4^First Department of Propaedeutic and Internal Medicine, Division of Diabetes, Laiko University Hospital, National and Kapodistrian University of Athens Medical School, Athens, Greece

## Abstract

**Background:**

Newer antidiabetic drugs, i.e., dipeptidyl peptidase-4 (DPP-4) inhibitors, sodium-glucose cotransporter-2 (SGLT-2) inhibitors, and glucagon-like peptide-1 receptor agonists (GLP-1 RAs) may exert distinct cardiovascular effects. We sought to explore their impact on vascular function.

**Methods:**

Published literature was systematically searched up to January 2018 for clinical studies assessing the effects of DPP-4 inhibitors, GLP-1 RAs, and SGLT-2 inhibitors on endothelial function and arterial stiffness, assessed by flow-mediated dilation (FMD) of the brachial artery and pulse wave velocity (PWV), respectively. For each eligible study, we used the mean difference (MD) with 95% confidence intervals (CIs) for FMD and PWV. The pooled MD for FMD and PWV were calculated by using a random-effect model. The presence of heterogeneity among studies was evaluated by the *I*^2^ statistic.

**Results:**

A total of 26 eligible studies (*n* = 668 patients) were included in the present meta-analysis. Among newer antidiabetic drugs, only SGLT-2 inhibitors significantly improved FMD (pooled MD 1.14%, 95% CI: 0.18 to 1.73, *p* = 0.016), but not DPP-4 inhibitors (pooled MD = 0.86%, 95% CI: -0.15 to 1.86, *p* = 0.095) or GLP-1 RA (pooled MD = 2.37%, 95% CI: -0.51 to 5.25, *p* = 0.107). Both GLP-1 RA (pooled MD = −1.97, 95% CI: -2.65 to -1.30, *p* < 0.001) and, to a lesser extent, DPP-4 inhibitors (pooled MD = -0.18, 95% CI: -0.30 to -0.07, *p* = 0.002) significantly decreased PWV.

**Conclusions:**

Newer antidiabetic drugs differentially affect endothelial function and arterial stiffness, as assessed by FMD and PWV, respectively. These findings could explain the distinct effects of these drugs on cardiovascular risk of patients with type 2 diabetes.

## 1. Introduction

Type 2 diabetes (T2D) is a chronic disease affecting 8.3% of the adult population worldwide, with a rising prevalence that renders its tackling a global challenge [1]. Patients with T2D are at high cardiovascular disease (CVD) risk [2, 3] and are characterized by micro- and macrovascular dysfunction which is of multifactorial origin [4, 5].

The safety and effects of newly licensed antidiabetic drugs on the cardiovascular system represent important clinical issues [6, 7]. Recent evidence from clinical trials suggests that newer antidiabetic drugs can not only exert glycemic-lowering properties but also decrease CVD risk [8, 9]. In this context, sodium-glucose cotransporter-2 (SGLT-2) inhibitors, i.e., empagliflozin in the Empagliflozin Cardiovascular Outcome Event Trial in Type 2 Diabetes Mellitus Patients-Removing Excess Glucose (EMPA-REG OUTCOME) study [8] and canagliflozin in the Canagliflozin Cardiovascular Assessment Study [10], significantly reduced the rates of CVD events, hospitalization for heart failure (HF), CVD, and total mortality, as well as improved kidney function in T2D patients with established CVD. Similar beneficial effects were reported for liraglutide, an once-daily glucagon-like peptide-1 receptor agonist (GLP-1 RA), and for semaglutide, an once-weekly GLP-1 RA, both of which reduced CVD morbidity and mortality (but not hospitalization for HF) in T2D patients with established CVD, in the Liraglutide Effect and Action in Diabetes: Evaluation of Cardiovascular Outcome Results (LEADER) trial [9] and the Trial to Evaluate Cardiovascular and Other Long-term Outcomes with Semaglutide in Subjects with Type 2 Diabetes (SUSTAIN-6) [11], respectively. In contrast, lixisenatide once daily and exenatide once weekly did not affect CVD risk in the Evaluation of Lixisenatide in Acute Coronary Syndrome (ELIXA) trial [12] and the Exenatide Study of Cardiovascular Event Lowering (EXSCEL) [13], respectively. Furthermore, dipeptidyl peptidase-4 (DPP-4) inhibitors seem to exert neutral effects on CVD risk as shown for alogliptin in the Examination of Cardiovascular Outcomes with Alogliptin versus Standard of Care (EXAMINE) trial [14] and for sitagliptin in the Trial Evaluating Cardiovascular Outcomes with Sitagliptin (TECOS) [15]. Saxagliptin was reported to increase the rate of hospitalization for HF [16] in the Saxagliptin Assessment of Vascular Outcomes Recorded in Patients with Diabetes Mellitus (SAVOR)–Thrombolysis in Myocardial Infarction (TIMI) 53 trial. Despite this evidence provided by large randomized clinical trials, the mechanisms by which antidiabetic drugs can affect CVD risk remain not entirely clear.

Vascular dysfunction is one of the initial steps in the atherosclerotic process [17, 18]. Endothelial function and arterial stiffness [17, 19] are two widely used indices of vascular function, which both offer prognostic information on the risk of CVD events in T2D patients [19]. Improvement of these indices represents one of the mechanisms by which drugs with established CVD benefits, such as statins, exert their effects [20, 21]. Currently, it remains unknown how newer antidiabetic drugs may affect vascular function as studies have yielded conflicting results.

We conducted a systematic review of the literature, followed by a meta-analysis, to investigate the effects of newer antidiabetic drugs, i.e., DPP-4 inhibitors, GLP-1 RAs, and SGLT-2 inhibitors, on vascular function as assessed by flow-mediated dilation (FMD) of the brachial artery and pulse wave velocity (PWV).

## 2. Patients and Methods

### 2.1. Literature Search

**Figure 1:**
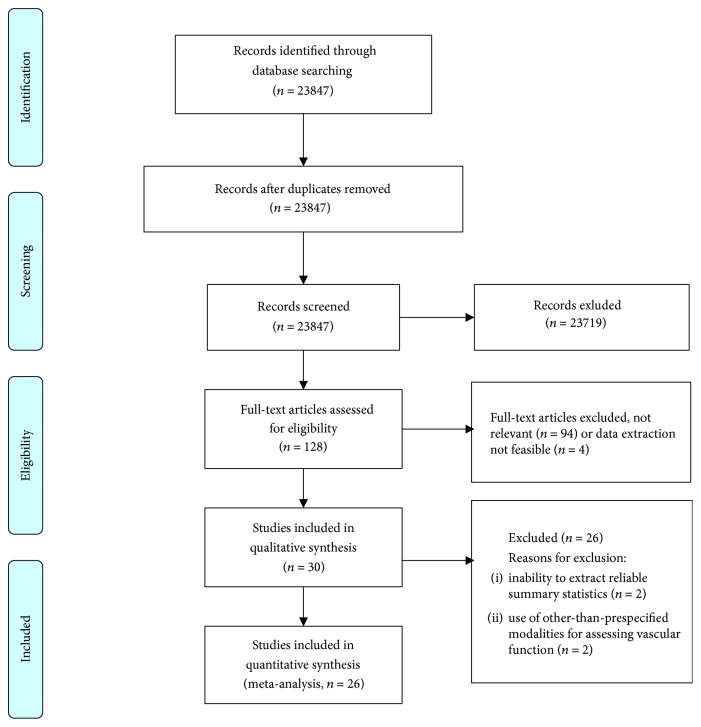
Study flow chart.

Eligible studies evaluating the effects of DPP-4 inhibitors, GLP-1 RAs, and SGLT-2 inhibitors on FMD and PWV were drawn from a systematic review of the English literature in the MEDLINE and Web of Science databases up to 31 January 2018. The medical terms (MeSH) used were the following: sodium-glucose cotransporter-2 OR SGLT2 OR empagliflozin OR canagliflozin OR dapagliflozin OR DPP-4 OR dipeptidyl peptidase-4 inhibitors OR sitagliptin OR saxagliptin OR vildagliptin OR linagliptin OR gemigliptin OR canagliptin OR teneligliptin OR alogliptin OR trelagliptin OR omarigliptin OR evogliptin OR dutogliptin OR GLP-1 OR glucagon-like peptide-1 OR exenatide OR lixisenatide OR dulaglutide OR liraglutide OR semaglutide AND endothelial function OR arterial stiffness OR flow-mediated dilation OR pulse wave velocity. Studies were also identified from searching the references of published articles. The PRISMA flow chart for the study is presented in Figure 1.

### 2.2. Study Eligibility

Studies were eligible if they were full-length publications in peer-reviewed journals reporting on (a) endothelium-dependent vasodilatory response by FMD and/or (b) arterial stiffness assessed by carotid-femoral, carotid-radial, or brachial-ankle PWV. Studies need also to be either double-blind, placebo-controlled, randomized clinical trials or observational studies assessing the outcomes of interest before and after treatment with a newer antidiabetic drug. No restriction criteria were imposed with regard to the size of the population studied. Analysis did not include studies assessing endothelial function or arterial stiffness by other markers. Only human studies were included in the analysis, whereas review articles were excluded.

### 2.3. Extraction of Data

Literature search, selection of studies, and extraction of data were performed independently by two investigators (KB and AA). Means and standard deviations (SD) of FMD and PWV as well as their changes following drug treatment were recorded from cumulative published data. In studies reporting standard error, SD was calculated using the equation SD = standard error∗√*n*. In studies reporting median and interquartile range, mean and SD were calculated and used in further analyses [22]. In those studies where extraction of cumulative statistics could not be reliably performed based on the full-length publication, a communication with the corresponding author was attempted to provide summary statistics [23–27].

### 2.4. Statistical Analysis

For each eligible study, we used the mean difference (MD) with 95% confidence intervals (CIs) for endothelium-dependent vasodilation and arterial stiffness as summary statistics (MD pre- and posttreatment). The difference in mean ± SD for FMD and PWV was included in the quantitative synthesis to explore the pooled MD after treatment with any of the studied antidiabetic drugs; the results were presented in respective forest plots. Subgroup analysis was performed for different drug classes. The presence of heterogeneity was evaluated by the *I*^2^ statistic. A random-effect model was used to obtain pooled MD and 95% CIs. Results were considered statistically significant at two-tailed *p* value < 0.05. The findings of the meta-analysis were also confirmed in leave-one-out sensitivity analysis. The quality of published studies was assessed by the modified version of the Downs and Black questionnaire [28]. The effect of publication bias on pooled estimates was explored by the Duval and Tweedie nonparametric “trim and fill” method and the construction of relevant funnel plots. All statistical analyses were performed by STATA software version 13.0 (StataCorp LLC, College Station, Texas, US).

## 3. Results

### 3.1. Qualitative Summary

The literature search identified 30 studies for potential inclusion in the present meta-analysis. Certain identified studies that were originally included in the qualitative synthesis had to be excluded from the quantitative synthesis due to not fulfilling the eligibility criteria. One study was excluded because it assessed endothelial function by reactive hyperemia peripheral arterial tonometry [29] and one because it used a 24 h approach to measure PWV [30]. Also, two studies had to be excluded due to inability to extract reliable summary statistics for the outcomes of interest [26, 27] (Figure 1). Therefore, a total of 26 studies (*n* = 668 patients) were finally included in the quantitative synthesis. The details of the individual studies included in the meta-analysis are summarized in Table 1.

All studies were published since 2012. The sample sizes of the studies ranged from 10 to 51 patients. The mean follow-up time was 152 days after initiation of treatment. The majority of studies were randomized controlled trials [23–25, 31–48], but some of them were uncontrolled or single-arm observational studies [49–53]. All studies performed the ultrasound-based technique to assess brachial artery FMD and PWV to assess arterial stiffness.

### 3.2. Quantitative Synthesis

#### 3.2.1. Effects of Newer Antidiabetic Drugs on Endothelial Function

**Figure 2:**
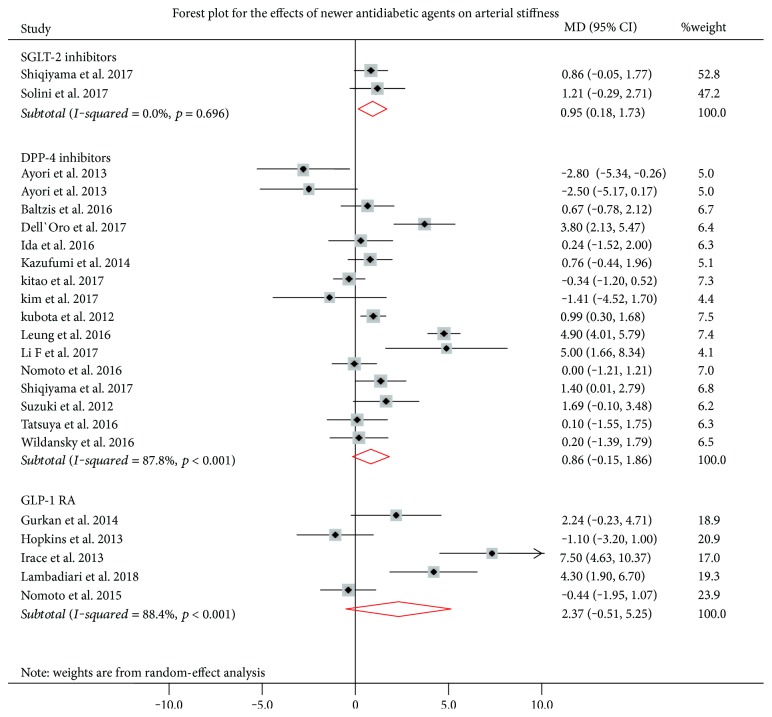
Effects of newer antidiabetic drugs on endothelial function. Squares indicate the mean difference (MD) and the respective 95% confidence intervals in flow-mediated dilatation (FMD) before/after treatment from eligible studies. The size of the squares corresponds to the weight of each study. The diamonds and their width represent the pooled MD and the 95% CI, respectively. DPP-4 = DPP-4 inhibitors, SGLT-2 inhibitors, and GLP-1 RAs and defines them under the figure. DPP-4: dipeptidyl peptidase-4; GLP-1 RAs: glucagon-like peptide-1 receptor agonists; SGLT-2: sodium-glucose cotransporter-2.

The effects of newer antidiabetic drugs on FMD are summarized in Figure 2. Overall, 16 studies investigated the effects of DPP-4 inhibitors on FMD. In the pooled meta-analysis, the effects of DPP-4 inhibitors on FMD was not statistically significant (pooled MD = 0.86%, 95% CI: -0.15 to 1.86, *p* = 0.095). But there was significant heterogeneity between studies (*I*^2^ = 87.8%, *p* < 0.0001). In leave-one-out sensitivity analysis, the results were unchanged (Table 2); only by excluding the study of Ayaori et al. [31] was a significant effect of DPP-4 on FMD observed (pooled MD = 1.25%, 95% CI: 0.24 to 2.27, *p* = 0.015), but heterogeneity among studies remained significant. Even within the group of DPP-4 inhibitors, no specific agent was associated with significant improvements in FMD.

For the effect of GLP-1 RAs (liraglutide and exenatide) on FMD (5 studies; *n* = 84 patients), the pooled estimate effect was not significant (pooled MD = 2.38%, 95% CI: -0.51 to 5.25, *p* = 0.107). Heterogeneity among studies was significant (*I*^2^ = 88.4%, *p* < 0.001). In leave-one-out sensitivity analysis, the results were unchanged and the heterogeneity among studies remained significant.

Only 2 eligible studies (*n* = 53 patients) evaluated the effects of SGLT-2 inhibitors on FMD. Dapagliflozin was the only SGLT-2 inhibitor used in these studies. SGLT-2 inhibitors increased FMD significantly (pooled MD = 0.95%, 95% CI: 0.18 to 1.73, *p* = 0.016).

In metaregression analysis, the mean difference in FMD was not associated with the size of the study (*b* = −0.033, *p* = 0.489), the duration of intervention (*b* = 0.002, *p* = 0.460), the age (*b* = −0.162, *p* = 0.163) or the sex (for males: *b* = 0.004, *p* = 0.931) of participants enrolled.

#### 3.2.2. Effects of Newer Antidiabetic Drugs on Arterial Stiffness

**Figure 3:**
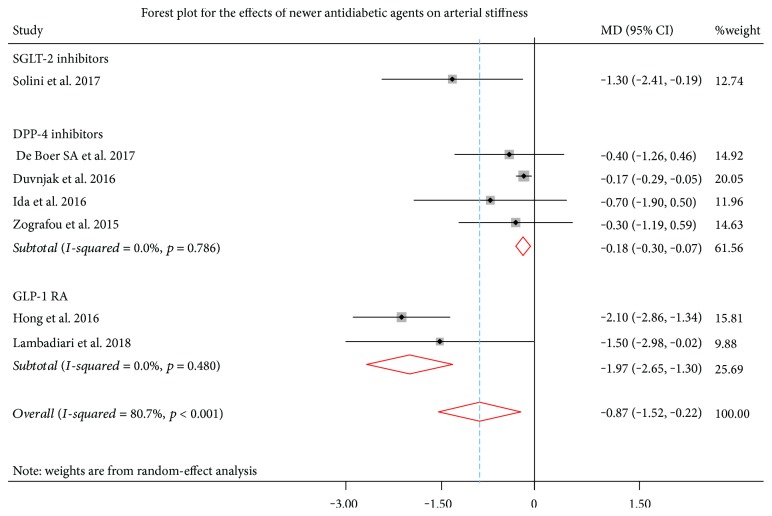
Effects of newer antidiabetic drugs on arterial stiffness. Squares indicate the mean difference (MD) and the respective 95% confidence intervals in pulse wave velocity (PWV) before/after treatment from eligible studies. The size of the squares corresponds to the weight of each study. The diamonds and their width represent the pooled weighted MD and the 95% CI, respectively. DPP-4: dipeptidyl peptidase-4; GLP-1 RAs: glucagon-like peptide-1 receptor agonists; SGLT-2: sodium-glucose cotransporter-2.

The effects of newer antidiabetic drugs on PWV are summarized in Figure 3. Marked heterogeneity was observed among analyzed studies (*I*^2^ = 80.7%, *p* < 0.001). A total of 4 studies (*n* = 132 patients) investigated the effects of DPP-4 inhibitors on arterial stiffness, which were associated with a significant reduction in PWV (pooled MD = −0.18, 95% CI: -0.30 to -0.07, *p* = 0.002). A similar effect was identified for GLP-1 RAs (2 studies; *n* = 62 patients), which were associated with a significant reduction in PWV (pooled MD = −1.97, 95% CI: -2.65 to -1.30, *p* < 0.001). For SGLT-2 inhibitors, only one study reported a reduction in PWV, and therefore, no safe conclusions can be drawn.

### 3.3. Quality Assessment of Included Studies and Publication Bias

**Figure 4:**
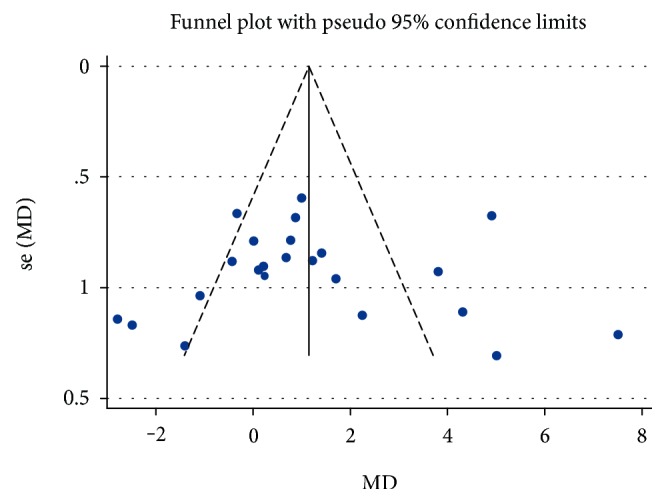
Funnel plot and assessment of publication bias. Funnel plot with 95% pseudoconfidence intervals of the effect size and its standard error for studies assessing the effects of newer antidiabetic drugs on the primary endpoint endothelial function. Large studies appear toward the top of the graph and tend to cluster near the mean effect size. Smaller studies appear toward the bottom of the graph and (since there is more sampling variation in effect size estimates in the smaller studies) will be dispersed across a range of values. The symmetric distribution of studies about the combined effect size indicates the absence of publication bias.

The quality of published studies was assessed by the modified Downs and Black checklist [28]. The quality of published studies is considered moderate with a mean modified Downs and Black score of 23.1. To explore publication bias, we constructed a funnel plot of published studies for the effect size of antidiabetic drugs on the primary endpoint of our study, i.e., endothelial function assessed by FMD. The funnel plot was symmetric, suggesting the absence of publication bias (Figure 4).

## 4. Discussion

In the present systematic review, we sought to explore the effects of newer antidiabetic drugs, namely, SGLT-2 inhibitors, DPP-4 inhibitors, and GLP-1 RAs, on vascular function. We hypothesized that the distinct profile of each antidiabetic drug class could be also associated with differences in their vascular effects. The systematic review of the published literature showed that evidence in this field is modest, based mainly on small randomized clinical trials with significant heterogeneity. In this context, published studies supported a beneficial effect of SGLT-2 on FMD, which seems to not be shared by GLP-1 RAs or DPP-4 inhibitors. Accordingly, evidence suggested a reduction in PWV by both DPP-4 inhibitors and GLP-1 RAs. These findings are potentially important as they suggest a different impact of newer antidiabetic drugs on vascular function, which could be linked with their distinct effects on CVD risk. However, these results should be interpreted with caution given the modest quality of evidence in the published literature and the significant heterogeneity between studies.

DPP-4 inhibitors are an antidiabetic drug class on which there is abundant clinical experience, since they have been marketed for over a decade (since 2006). Large randomized clinical trials in the field are consistent in their findings and support a neutral effect of DPP-4 inhibitors on CVD outcomes. However, there is evidence that saxagliptin may increase the risk for HF hospitalization [16]. Recent meta-analyses have also found that DPP-4 inhibitors do not affect the risk for CVD mortality and stroke [54]. Data from the small clinical studies included in the present meta-analysis indicate a marginal effect of DPP-4 drugs on FMD and a significant reduction in PWV. These small effects could be related to the glucose-lowering properties of DPP-4 inhibition but may not be commonly shared by all agents of the DPP-4 drug class. This is an interest finding which (a) confirms the safety profile of this drug class and (b) may explain the neutral effect of certain DPP-4 drugs on CVD outcomes.

For SGLT-2 inhibitors, evidence suggests a beneficial effect of these agents on CVD risk and mortality in T2D patients with established CVD. Evidence from randomized clinical trials suggests that empagliflozin and canagliflozin significantly reduce the CVD morbidity, all-cause mortality, and CVD mortality as well as HF hospitalization and nephropathy development or progression [8]. Similar effects have also been reported for canagliflozin [10]. These benefits could be related to glucose-lowering as well as to reductions in blood pressure, weight, and serum uric acid and to improvements in oxidative stress, glomerular hyperfiltration, albuminuria, arterial stiffness, plasma lipids, sympathetic nervous system activity, myocardial oxygen consumption, and cardiac workload [55]. Our findings complement these SGLT-2 inhibitor actions, suggesting also a significant improvement in FMD. Concerning the impact of SGLT-2 inhibitors on arterial wave velocity, it should be noted that despite the ample evidence on the impact of this class of drugs on natriuresis and blood pressure [8, 10], more data are required to assess the effects of these drugs on PWV, as there are only few published data on this topic [46].

Our findings also agree with the published evidence from large randomized clinical trials on the effects of liraglutide [9] and semaglutide [11] on CVD outcomes in T2D patients. In the present meta-analysis, GLP-1 RAs significantly decreased PWV in T2D patients, although they did not affect FMD. Since the effects of these drugs on CVD risk is still debatable [12, 56], it remains to be seen whether their impact on vascular function may play a role in determining their CVD effects.

The limitations of the existing studies in the field should be noted. The published evidence is modest and mainly based on small-sized randomized clinical studies, some of which were uncontrolled. Furthermore, studies were significantly heterogeneous, and therefore, the results of the present meta-analysis should be interpreted cautiously. The number of the published studies in this field did not allow for subgroup analysis per drug class or for between-agent comparisons within the same drug class. Furthermore, based on the published literature, we cannot conclude whether beneficial effects of SGLT-2 inhibitors, DDP-4 inhibitors, and GLP-1 RAs are due to direct glucose-lowering effects or to indirect effects driven by the modulation of other cardiovascular risk factors such as body weight loss and arterial blood pressure modification [8–11, 57]. More data is also warranted for the effect of SGLT-2 inhibitors on arterial stiffness as well as endothelial function as there are limited published studies reporting on their effects.

## 5. Conclusion

In conclusion, the present meta-analysis suggests that the published literature in the field of newer antidiabetic drugs and vascular function is of modest quality and characterized by significant heterogeneity among studies. Overall, both DPP-4 inhibitors and GLP-1 RAs were shown to significantly decrease PWV without affecting FMD. In contrast, SGLT-2 inhibitors significantly improved FMD, but concrete data on their effects on PWV is still missing. Whether these distinct properties of newer antidiabetic drugs, in relation to their effects on endothelial function and arterial stiffness, may explain their differential effects on CVD risk remains to be elucidated in future studies.

## Figures and Tables

**Table 1:** Summary characteristics of studies included in the analysis.

Author	Class	Agent	Study design	*N*	Duration	FMD (%)		PWV (m/s)	
					(days)	Baseline	Post	Baseline	Post
Ayaori et al. [31]	DPP-4i	Sitagliptin	RCT	42	42	7.2 ± 5.9	4.4 ± 5.9		
		Alogliptin				6.9 ± 6.3	4.4 ± 6.2		
Baltzis et al. [32]	DPP-4i	Linagliptin	RCT	19	84	6.5 ± 2.1	7.2 ± 2.5		
de Boer et al. [33]	DPP-4i	Linagliptin	RCT	22	182			8.7 ± 1.6	8.3 ± 1.3
Dell'Oro et al. [34]	DPP-4i	Saxagliptin	RCT	16	360	3.6 ± 0.3	7.4 ± 0.8		
Duvnjak et al. [49]	DPP-4i	Sitagliptin/vildagliptin	Open label (NR)	51	90			8.6 ± 0.3	8.4 ± 0.3
Gurkan et al. [35]	GLP-1 RA	Exenatide	RCT	17	182	6.4 ± 5.7	8.6 ± 4.7		
Hong et al. [50]	GLP-1 RA	Exenatide	Single arm (NR)	32	90			7.2 ± 2.2	5.1 ± 0.1
Hopkins et al. [51]	GLP-1 RA	Exenatide/liraglutide	Single arm (NR)	11	180	6.2 ± 2.3	5.1 ± 2.7		
Ida et al. [52]	DPP-4i	Trelagliptin	Single arm (NR)	27	84	2.4 ± 2.7	2.7 ± 3.8	16.3 ± 2.4	15.6 ± 2.2^∗^
Irace et al. [36]	GLP-1 RA	Exenatide	RCT	10	112	1.6 ± 2.9	9.1 ± 3.6		
Nakamura et al. [24]	DPP-4i	Sitagliptin	RCT	24	90	5.4 ± 2.3	6.2 ± 2.0		
Kim et al. [37]	DPP-4i	Vildagliptin	RCT	17	84	9.4 ± 5.0	7.9 ± 4.3		
Kitao et al. [38]	DPP-4i	Vildagliptin	RCT	48	84	5.5 ± 2.0	5.1 ± 2.3		
Kubota et al. [53]	DPP-4i	Sitagliptin	Single arm (NR)	40	90	4.1 ± 1.5	5.1 ± 1.6		
Lambadiari et al. [39]	GLP-1 RA	Liraglutide	RCT	30	180	8.9 ± 3.0	13.2 ± 6.0	11.8 ± 2.5	10.3 ± 3.3
Leung et al. [40]	DPP-4i	Sitagliptin/vildagliptin	RCT	25	365	2.4 ± 1.6	7.3 ± 1.6		
Li et al. [41]	DPP-4i	Saxagliptin	RCT	14	84	9.3 ± 4.7	14.3 ± 4.3		
Nomoto et al. [42]	DPP-4i	Sitagliptin	RCT	48	182	5.6 ± 2.8	5.6 ± 2.8		
Nomoto et al. [43]	GLP-1 RA	Liraglutide	RCT	16	98	6.0 ± 2.6	5.6 ± 1.6		
Shigiyama et al. [44]	SGLT-2i	Dapagliflozin	RCT	37	112	4.8 ± 1.9	5.7 ± 2.1		
Shigiyama et al. [45]	DPP-4i	Linagliptin	RCT	29	112	4.9 ± 2.7	6.3 ± 2.7		
Solini et al. [46]	SGLT-2i	Dapagliflozin	RCT	16	2	2.8 ± 2.3	4.0 ± 2.1	10.1 ± 1.6	8.9 ± 1.6
Suzuki et al. [25]	DPP-4i	Sitagliptin	RCT	12	90	3.7 ± 2.3	5.4 ± 2.2		
Maruhashi et al. [23]	DPP-4i	Sitagliptin	RCT	17	720	4.3 ± 2.6	4.4 ± 2.3		
Widlansky et al. [47]	DPP-4i	Saxagliptin	RCT	16	56	5.6 ± 2.3	5.8 ± 2.3		
Zografou et al. [48]	DPP-4i	Vildagliptin	RCT	32	180			8.6 ± 2.1	8.3 ± 1.5

DPP-4i: dipeptidyl peptidase-4 inhibitor; FMD: flow-mediated dilation; GLP-1 RA: glucagon-like peptide-1 receptor agonist; NR: nonrandomized; RCT: randomized clinical trial; SGLT-2i: sodium-glucose cotransporter-2 inhibitor; PWV: pulse wave velocity. *N* refers to the active treatment group. The full list of references of the studies included in the table is provided in the supplementary material. ^∗^Measured as brachial-ankle PWV.

**Table 2:** Leave-one-out sensitivity analysis for the effects of newer antidiabetics on endothelial function.

Study excluded	MD (95% CI)	*p* value	Heterogeneity (*I*^2^)
*DPP-4 inhibitors*			
Ayaori et al. [31]	1.253 (0.242 to 2.265)	*p* = 0.015	87.7%, *p* < 0.001
Baltzis et al. [32]	0.866 (-0.205 to 1.936)	*p* = 0.113	88.6%, *p* < 0.001
Dell'Oro et al. [34]	0.658 (-0.365 to 1.682)	*p* = 0.207	87.8%, *p* < 0.001
Ida et al. [52]	0.897 (-0.151 to 1.945)	*p* = 0.095	88.5%, *p* < 0.001
Nakamura et al. [24]	0.858 (-0.226 to 1.943)	*p* = 0.121	88.6%, *p* < 0.001
Kim et al. [37]	0.962 (-0.066 to 1.990)	*p* = 0.067	88.3%, *p* < 0.001
Kitao et al. [38]	0.948 (-0.115 to 2.011)	*p* = 0.080	87.1%, *p* < 0.001
Kubota et al. [53]	0.832 (-0.329 to 1.993)	*p* = 0.160	88.6%, *p* < 0.001
Leung et al. [40]	0.566 (-0.130 to 1.261)	*p* = 0.111	68.6%, *p* < 0.001
Li et al. [41]	0.679 (-0.337 to 1.695)	*p* = 0.190	88.1%, *p* < 0.001
Nomoto et al. [42]	0.917 (-0.153 to 1.986)	*p* = 0.093	88.2%, *p* < 0.001
Shigiyama et al. [44]	0.813 (-0.262 to 1.888)	*p* = 0.138	88.6%, *p* < 0.001
Suzuki et al. [25]	0.798 (-0.261 to 1.858)	*p* = 0.140	88.6%, *p* < 0.001
Maruhashi et al. [23]	0.906 (-0.152 to 1.964)	*p* = 0.093	88.4%, *p* < 0.001
Widlansky et al. [47]	0.899 (-0.161 to 1.960)	*p* = 0.097	88.5%, *p* < 0.001
*GLP-1 RA*			
Gurkan et al. [35]	2.435 (-1.177 to 6.047)	*p* = 0.186	91.2%, *p* < 0.001
Hopkins et al. [51]	3.275 (-0.132 to 6.682)	*p* = 0.060	89.2%, *p* < 0.001
Irace et al. [36]	1.141 (-1.194 to 3.476)	*p* = 0.338	80.2%, *p* = 0.001
Lambadiari et al. [39]	1.902 (-1.399 to 5.203)	*p* = 0.259	89.3%, *p* < 0.001
Nomoto et al. [42]	3.155 (-0.376 to 6.687)	*p* = 0.080	88.1%, *p* < 0.001

DPP-4: dipeptidyl peptidase-4 inhibitors; GLP-1 RA: glucagon-like peptide-1 receptor agonists.
